# Prediction of sepsis-related outcomes in neonates through systematic genotyping of polymorphisms in genes for innate immunity and inflammation: a narrative review and critical perspective

**DOI:** 10.1590/1516-3180.2013.1315519

**Published:** 2013-10-01

**Authors:** Juliana Kilesse Carvalho, Daniella Batalha Moore, Ricardo Alves Luz, Pedro Paulo Xavier-Elsas, Maria Ignez Capella Gaspar-Elsas

**Affiliations:** I MD, MSc. Neonatologist, Laboratory of Human Pathophysiology, Department of Pediatrics, Instituto Fernandes Figueira (IFF), Fundação Oswaldo Cruz (Fiocruz), Rio de Janeiro, Brazil.; II MD, MSc, PhD. Associate Professor, Discipline of Clinical Immunology, Department of Internal Medicine, Universidade Federal Fluminense (UFF), Niterói, Brazil.; III MSc, PhD. Research associate, Department of Immunology, Instituto de Microbiologia Paulo de Góes (IMPG), Universidade Federal do Rio de Janeiro (UFRJ), Rio de Janeiro, Brazil.; IV MD, MSc, PhD. Associate Professor, Department of Immunology, Instituto de Microbiologia Paulo de Góes (IMPG), Universidade Federal do Rio de Janeiro (UFRJ), Rio de Janeiro, Brazil.; V MD, PhD. Head of the Laboratory of Human Pathophysiology, Department of Pediatrics, Instituto Fernandes Figueira (IFF), Fundação Oswaldo Cruz (Fiocruz), Rio de Janeiro, Brazil.

**Keywords:** Neonatology, Sepsis, Cytokines, Genetic predisposition to disease, Polymorphism, genetic, Neonatologia, Sepse, Citocinas, Predisposição genética para doença, Polimorfismo genético

## Abstract

**CONTEXT AND OBJECTIVE:**

Neonatal sepsis is associated with premature birth and maternal infection. Large-scale studies seek to define markers that identify neonates at risk of developing sepsis. Here, we examine whether the scientific evidence supports systematic use of polymorphism genotyping in cytokine and innate immunity genes, to identify neonates at increased risk of sepsis.

**DESIGN AND SETTING:**

Narrative literature review conducted at Fernandes Figueira Institute, Brazil.

**METHODS:**

The literature was searched in PubMed, Embase (Excerpta Medica Database), Lilacs (Literatura Latino-Americana e do Caribe em Ciências da Saúde), SciELO (Scientific Electronic Library Online) and Cochrane Library. From > 400,000 references, 548 were retrieved based on inclusion/exclusion criteria; 22 were selected for detailed analysis after quality assessment.

**RESULTS:**

The studies retrieved addressed the impact of gene polymorphisms relating to immune mechanisms (most often TNF-a, LT-a, IL-6, IL-1β, IL-1ra, L-selectin, CD14 and MBL) or inflammatory mechanisms (ACE and angiotensin II receptors; secretory PLA2; and hemostatic factors). Despite initial reports suggesting positive associations between specific polymorphisms and increased risk of sepsis, the accumulated evidence has not confirmed that any of them have predictive power to justify systematic genotyping.

**CONCLUSIONS:**

Sepsis prediction through systematic genotyping needs to be reevaluated, based on studies that demonstrate the functional impact of gene polymorphisms and epidemiological differences among ethnically distinct populations.

## Abstract (pt)

**CONTEXTO E OBJETIVO:**

A sepse neonatal está associada ao parto prematuro e à infecção materna. Estudos em grande escala buscam marcadores que identifiquem neonatos em risco de desenvolver sepse. Examinamos aqui se a evidência científica apoia o uso sistemático de genotipagem dos polimorfismos em genes de citocinas e imunidade inata, para identificar neonatos com risco elevado de sepse.

**TIPO DE ESTUDO E LOCAL:**

Revis ão narrativa da literatura, Instituto Fernandes Figueira, Brasil. M ÉTODOS: Busca online da literatura foi feita no PubMed, Embase (Excerpta Medica Database), Lilacs (Literatura Latino-Americana e do Caribe em Ciências da Saúde), SciELO (Scientific Electronic Library Online) e Cochrane Library. De mais de 400.000 referências, 548 foram recuperadas com base nos critérios de inclusão/exclusão, e 22, selecionadas para análise detalhada após verificação da qualidade.

**RESULTADOS:**

Recuperamos estudos de impacto dos polimorfismos em genes relacionados com mecanismos imunes (mais frequentemente, TNF-a, LT-a, IL-6, IL-1 β, IL-1ra, L-selectin, CD14, e MBL) ou inflamatórios (ACE e receptores de angiotensina II; PLA2 secretória; fatores hemostáticos). Contrariando estudos que inicialmente sugeriram associação positiva entre polimorfismos específicos e risco aumentado de sepse, a evidência acumulada não confirmou, para qualquer deles, valor preditivo que justifique genotipagem sistemática para orientar antibioticoterapia.

**CONCLUSÕES:**

A previsão da sepse por meio de genotipagem sistemática precisa ser reavaliada, com base em estudos que demonstram o impacto funcional de polimorfismos gênicos e as diferenças epidemiológicas entre populações etnicamente distintas.

## INTRODUCTION

Neonatal sepsis, defined by systemic circulatory abnormalities (predominantly peripheral vasoconstriction, oliguria and ischemic damage to inner organs) and a variable spectrum of clinical signs, resulting from invasion of the bloodstream by bacteria and other pathogens, as well as from the ineffective host response, in infants up to their first month of life. ^1^

It results in one million deaths each year (42% in the first week of life). This amounts to 10% of all mortality under the age of five years. ^2^ In Brazil, neonatal sepsis is a leading cause of mortality during the neonatal period, which represents an estimated 60% of all childhood mortality. ^3^

Many of the clinical problems in recognition and management of neonatal sepsis are related to the complex biology of the neonatal period, when the physiological immaturity of the immune system and the fragility of the skin and mucosal barriers can be accompanied by a variety of additional risk factors. Neonatal sepsis is defined by: a) differential exposure to specific classes of infectious pathogens; b) the additional impacts of prematurity, low birth weight (LBW) and very low birth weight (VLBW); c) exposure to use of immunomodulatory drugs; and d) risks associated with therapeutic procedures in neonatal intensive care units. ^4-^ ^)(^ ^9^

Diversity of infectious exposures is relevant to the clinical differences between early-onset and late-onset neonatal sepsis. The former is classically associated with pathogens present at the fetal-maternal interface, ^10^ while the latter (much more frequent) reflects invasion from nosocomial microorganisms, with a risk that increases with the length of hospitalization and invasiveness of procedures, and when microbiological control is suboptimal. ^9^ ^,^ ^11^ ^,^ ^12^ The risks of neonatal infection and sepsis are inversely proportional to gestational age, thus suggesting that critical components of the immune system reach maturity only in the final weeks of gestation, ^13^ ^,^ ^14^ just before the newborn needs them to successfully manage the transition from microbiological sterility to colonization by a healthy microbiota. It remains to be established whether the increase in birth weight as gestational age approaches 37 weeks of gestation plays any additional role in protecting the neonate from infection. ^14^

The variable of clinical evolution of neonatal sepsis is related to individual variability in immune competence, especially with regard to innate immunity, ^15^ and to differences in responses to therapy. It is difficult to define which infants are at greatest risk of developing sepsis, among those exposed to similar environmental challenges. Even after sepsis has already developed, it remains difficult to monitor it through specific laboratory criteria, since pathogen isolation from blood and other sites is limited by low sensitivity and by the limited amounts that can be sampled. Aggressive antibiotic prophylaxis, on the other hand, significantly increases the risk of drug toxicity in premature infants, who lack efficient renal and hepatic detoxification mechanisms. Consequently, there is still a lack of tests for ensuring early and reliable diagnosing of neonatal sepsis, and for defining the patients who are most likely to benefit from aggressive antimicrobial treatment. ^16^ ^,^ ^17^

A comprehensive review by Härtel et al. ^18^ addressed the complexities involved in defining the individual differences that influence the risk of neonatal sepsis and related outcomes, especially with regard to specific genes of relevance to the host-pathogen relationship and to the collective influence of gene pools that differ among ethnically diverse populations. These authors acknowledged that host genetic markers with prognostic value for sepsis might be helpful in managing the neonates who would be most likely to benefit from aggressive antimicrobial therapy, and they put the challenges involved into perspective. In addition to the technical difficulties inherent to all genotyping studies, the diversity of candidate genes, multiplicity of existing alleles at each locus and variable locations of polymorphisms in promoter regions, exons and introns, as well as their highly unpredictable functional impact, must be taken into account. By definition, no single locus is thought to control susceptibility to neonatal sepsis, but a number of candidate genes have been studied because their biological effects that have been demonstrated or predicted would be consistent with a plausible pathogenetic mechanism.

This is the case of genes expressed in the context of innate immunity and inflammation, including: a) cytokines and related regulatory factors (TNF-a, LT-a, IL-6, IL-1β and IL-1ra); b) microbial recognition receptors (CD14, *toll-like* receptors and nucleotide-binding oligomerization domains/NOD); c) microbicidal proteins (mannose-binding lectin/MBL); and d) systemic effectors of inflammation in general (secretory phospholipase A2/PLA2, angiotensin/AT-converting enzyme/ACE and ATII receptors, and factors involved in hemostasis).

The review by Härtel et al. emphasized the need for: a) reproduction of the initial results in larger cohorts; b) adherence to rigorous definitions of sepsis and related outcomes; and c) adequate control over the effects of ethnic variation. ^18^ However, these authors did not issue specific recommendations for systematic screenings for any of the above genes in the context of neonatal sepsis. Because of the significant number of studies published since their original review of this field, we have reassessed here whether the available evidence warrants such recommendations.

## OBJECTIVE

This study aimed to analyze whether the available scientific evidence supports systematic use of genotyping of one or more polymorphisms in cytokine and innate immunity genes as a means of identifying neonates who are at increased risk of sepsis. 

## METHODS

We conducted an online review of the literature, and followed the guidelines provided by Preferred Reporting Items for Systematic reviews and Meta-Analyses (PRISMA). We searched the scientific literature for published studies that support use of genotyping for single nucleotide polymorphisms (SNPs) in newborns as biomarkers associated with the risk of sepsis and/or its complications. The selection of studies for detailed analysis was carried out independently by two investigators, who had received training in Allergy and Clinical Immunology and had professional experience in Neonatology. The steps in retrieval and analysis of the literature were as follows:

### 1. Identification of primary studies

We carried out a systematic search of electronic databases for published studies, primarily in PubMed Medline, but also including SciELO (Scientific Electronic Library Online), Embase (Excerpta Medica Database) and, through the Virtual Health Library (BVS), the Lilacs (Literatura Latino-Americana e do Caribe em Ciências da Saúde) and Cochrane Library databases. We did not search other databases that include unpublished studies, dissertations and theses, or research projects. The search of the literature was based on the PICO strategy, which is an acronym for the following: P (patient) - newborns aged 0-28 months, without restriction on studies between term and preterm, or between LBW and VLBW; I (intervention) - the studies included conducted genotyping of polymorphisms in order to evaluate associations with the risk of neonatal sepsis; C (comparison) - comparison between groups with and without neonatal sepsis, correlating with the genetic polymorphisms found; and O (outcome) - neonatal sepsis.

The following Medical Subject Headings (MeSH) subheadings were used: "neonate or premature or VLBW or LBW"; "sepsis or neonatal infections"; "polymorphism or polymorphisms or genetic variability or polymorphism or genotype". The search strategy is summarized in Table 1.

**Table 1:** Search strategy

Electronic search	Database	Filters	Search terms	Results
Search through NCBI (http://www.ncbi.nlm.nih.gov/pubmed)	PubMed	Limits: human, newborn, case control studies	(neonate OR premature OR VLBW OR LBW) AND (sepsis OR neonatal infections) AND (polymorphism OR polymorphisms OR genetic variability OR genotype)	Retrieved articles: 548Excluded articles: 526Included articles: 22
Search through Bireme-BVS (http://regional.bvsalud.org)	Lilacs (Literatura Latino-Americana e do Caribe em Ciências da Saúde)	Limits: human, newborn Major subject: genetic polymorphism		
	Cochrane Library	No limits		
Search though Embase Biomedical Answers (http://embase.periodicos.saude.gov.br)	Embase (Excerpta Medica Database)	Limits: human, newborn infection		
Search though SciELO (http://www.scielo.org)	SciELO (Scientific Electronic Library Online)	No limits		
Last search date: September 18, 2012				

VLBW = very low birth weight; LBW = low birth weight.

Our latest search was finished in September 2012. References in English, Spanish and Portuguese were analyzed, while articles in other languages for which only the abstract could be obtained in English were excluded.

### 2. Study selection for analysis 

Case-control studies evaluating the impact of genetic variability on sepsis in newborns were included. The patient group was composed of septic newborns and the control group was composed of healthy newborns or non-septic newborns. The relevant outcome was neonatal sepsis. 

We excluded the following: case reports; studies that did not evaluate sepsis as an outcome; methodological reviews and technical studies; studies focusing on children outside of the neonatal age group; studies on genetic diversity of pathogens rather than on patients; and any other papers retrieved that did not match the inclusion criteria. 

The abstracts of all studies identified in the original search after combining the descriptors were evaluated. After elimination of all papers that failed to meet the inclusion criteria, the manuscripts were fully analyzed. 

All the papers that were subjected to in-depth analysis had been retrieved through the primary PubMed search, even though some of them were also retrieved through the complementary searches in Embase, Lilacs, SciELO and the Cochrane Library. 

### 3. Quality assessment

Three of the co-authors (Moore DB, Gaspar-Elsas MI and Carvalho JK) independently applied the previously published methodological standards of Bogardus et al. ^19^ for assessing the quality of molecular genetic studies within the field of clinical epidemiology, following the recommendations of Peters et al. ^20^ A total of 22 papers were evaluated with regard to the following methodological standards: reproducibility, objectivity, case definition, adequacy of case group spectrum, control group definition, adequacy of the control group and quantitative summary (Table 2). ^9^ ^,^ ^21^ ^-^ ^41^ We further assessed the quality of the studies by means of the checklist of Downs and Black (Table 3). ^42^ Exclusions were always based on noncompliance with the criteria of the ideal methodological standard. In addition, every study was further examined for possible methodological flaws and interpretation of findings. ^43^

**Table 2:** Evaluation of methodological quality using Bogardus method

Study	1	2	3	4	5	6	7
Aydemir et al.^21^	no	yes	yes	yes	yes	no	yes
Härtel et al.^9^	no	yes	yes	yes	yes	no	yes
Abu-Maziad et al.^22^	no	yes	yes	yes	yes	no	no
Auriti et al.^23^	no	yes	yes	yes	yes	no	yes
Koroglu et al.^24^	no	yes	yes	yes	yes	no	yes
Spiegler et al.^25^	no	yes	yes	yes	yes	no	yes
Abdel-Hady et al.^26^	no	yes	yes	yes	yes	no	yes
Bertalan et al.^27^	no	yes	yes	yes	yes	no	no
Reiman et al.^28^	no	yes	yes	yes	yes	no	yes
Dzwonek et al.^29^	no	yes	yes	yes	yes	no	yes
van Der Zwet et al.^30^	no	yes	yes	no	no	no	yes
Schueller et al.^31^	no	yes	yes	yes	no	yes	yes
Derzbach et al.^32^	no	yes	yes	yes	yes	no	no
Härtel et al.^33^	no	yes	yes	yes	yes	no	yes
Baier et al.^34^	no	yes	yes	yes	yes	yes	yes
Göpel et al.^35^	no	yes	yes	yes	yes	no	yes
John Baier et al.^36^	no	yes	yes	yes	yes	no	no
Hedberg et al.^37^	no	yes	yes	yes	yes	no	no
Ahrens et al.^38^	no	yes	yes	yes	yes	no	yes
Bessler et al.^39^	no	yes	yes	yes	yes	no	yes
Harding et al.^40^	yes	yes	yes	yes	yes	yes	yes
Treszl et al.^41^	no	yes	yes	yes	yes	no	yes

1 = reproducibility; 2 = definition of cases; 3 = adequacy of cases; 4 = definition of control; 5 = adequacy of control; 6 = objectivity; 7 = quantification of results.

**Table 3:** Assessment of study quality by means of the Downs and Black checklist^42^

Questions (Q)		Aydemir et al.^21^		Härtel et al.^9^		Abu-Maziad et al.^22^		Auriti et al.^23^		Koroglu et al.^24^		Spiegler et al.^25^		Abdel-Hady et al.^26^		Bertalan et al.^27^		Reiman et al.^28^		Dzwonek et al.^29^		van Der Zwet et al.^30^		Schueller et al.^31^		Derzbach et al.^32^		Härtel et al.^33^		Baier et al.^34^		Göpel et al.^35^		John Baier et al.^36^		Hedberg et al.^37^		Ahrens et al.^38^		Bessler et al.^39^		Harding et al.^40^		Treszl et al.^41^
Q 1		yes		yes		yes		yes		yes		yes		yes		yes		yes		yes		yes		yes		yes		yes		yes		yes		yes		yes		yes		yes		yes		yes
Q 2		yes		yes		yes		yes		yes		yes		yes		yes		yes		yes		yes		yes		yes		yes		yes		yes		yes		yes		yes		yes		yes		yes
Q 3		yes		yes		yes		yes		yes		yes		yes		yes		yes		yes		yes		yes		yes		yes		yes		yes		yes		yes		yes		yes		yes		yes
Q 4		yes		yes		yes		yes		yes		yes		yes		yes		yes		yes		yes		yes		yes		yes		yes		yes		yes		yes		yes		yes		yes		yes
Q 5		yes		yes		yes		yes		yes		yes		yes		yes		yes		yes		yes		yes		yes		yes		yes		yes		yes		yes		yes		yes		yes		yes
Q 6		yes		yes		yes		yes		yes		yes		yes		yes		yes		yes		yes		yes		yes		yes		yes		yes		yes		yes		yes		yes		yes		yes
Q 10		no		yes		yes		yes		yes		yes		yes		yes		yes		yes		no		yes		yes		yes		yes		yes		yes		yes		yes		yes		yes		no
Q 11		no		no		no		yes		no		no		no		no		no		no		yes		no		no		no		no		no		no		no		no		no		no		no
Q 13		yes		yes		yes		yes		yes		yes		yes		yes		yes		yes		yes		yes		yes		yes		yes		yes		yes		yes		yes		yes		yes		yes
Q 14		no		no		no		no		no		no		no		no		no		no		no		yes		no		no		yes		no		no		no		no		no		yes		no
Q 15		no		no		no		no		no		no		no		no		no		no		no		yes		no		no		yes		no		no		no		no		no		yes		no
Q 18		yes		yes		yes		yes		yes		yes		yes		yes		yes		yes		yes		yes		yes		yes		yes		yes		yes		yes		yes		yes		yes		yes
Q 20		yes		yes		yes		yes		yes		yes		yes		yes		yes		yes		yes		yes		yes		yes		yes		yes		yes		yes		yes		yes		yes		yes
Q 21		yes		yes		yes		yes		yes		yes		yes		yes		yes		yes		yes		no		yes		yes		yes		yes		yes		yes		yes		yes		yes		yes
Q 22		yes		yes		yes		yes		yes		yes		yes		yes		yes		yes		yes		yes		yes		yes		yes		yes		yes		yes		yes		yes		yes		yes
Q 25		no		yes		yes		yes		yes		yes		yes		yes		yes		yes		yes		no		yes		yes		yes		yes		yes		yes		yes		yes		yes		yes
Q 27		no		yes		yes		no		yes		no		no		no		yes		no		no		no		no		yes		yes		no		no		no		yes		no		yes		no

Down and Black checklist for assessing study quality. Q 1 = Is the hypothesis/aim/objective of the study clearly described?; Q 2 = Are the main outcomes to be measured clearly described in the Introduction or Methods section?; Q 3 = Are the characteristics of the patients included in the study clearly described?; Q 4 = Are the interventions of interest clearly described?; Q 5 = Are the distributions of principal confounders in each group of subjects to be compared clearly described?; Q 6 = Are the main findings of the study clearly described?; Q 7 = Does the study provide estimates of the random variability in the data for the main outcomes?; Q 8 = Have all important adverse events that may be a consequence of the intervention been reported?; Q 9 = Have the characteristics of patients lost to follow-up been described? Q 10 = Have actual probability values been reported (e.g. 0.035 rather than < 0.05) for the main outcomes except where the probability value is less than 0.001?; Q 11 = Were the subjects asked to participate in the study representative of the entire population from which they were recruited?; Q 12 = Were those subjects who were prepared to participate representative of the entire population from which they were recruited?; Q 13 = Were the staff, places, and facilities where the patients were treated, representative of the treatment the majority of patients receive?; Q 14 = Was an attempt made to blind study subjects to the intervention they have received?; Q 15 = Was an attempt made to blind those measuring the main outcomes of the intervention?; Q 16 = If any of the results of the study were based on "data dredging", was this made clear?; Q 17 = In trials and cohort studies, do the analyses adjust for different lengths of follow-up of patients, or in case-control studies, is the time period between the intervention and outcome the same for cases and controls?; Q 18 = Were the statistical tests used to assess the main outcomes appropriate?; Q 19 = Was compliance with the intervention/s reliable?; Q 20 = Were the main outcome measurements used accurate (valid and reliable)?; Q 21 = Were the patients in different intervention groups (trials and cohort studies) or were the cases and controls (case-control studies) recruited from the same population?; Q 22 = Were study subjects in different intervention groups (trials and cohort studies) or were the cases and controls (case-control studies) recruited over the same period of time?; Q 23 = Were study subjects randomized to intervention groups?; Q 24 = Was the randomized intervention assignment concealed from both patients and healthcare staff until recruitment was complete and irrevocable?; Q 25 = Was there adequate adjustment for confounding in the analyses from which the main findings were drawn?; Q 26 = Were losses of patients from follow-up taken into account?; Q 27 = Did the study have sufficient power to detect a clinically important effect where the probability value for a difference being due to chance is less than 5%? Note: Questions 7, 8, 9, 12, 16, 17, 19, 23, 24 and 26 were considered to be not applicable to the type of studies included in this in-depth review.

### 4. Data analysis 

In this step, we aimed to define whether the conclusions in each paper did or did not support the assumption that genotyping of polymorphisms of interest is well-established as a laboratory test for systematic evaluation of the risk of neonatal sepsis and its complications. Data from each of the selected references that were used for this definition were summarized in a descriptive form, excluding technical details and quantitative data, but recording the nature of the evidence, clarity of the measurements, adequacy of the methods, information on potential conflicts of interest and coherence between the actual data and the conclusions and/or recommendations. 

## RESULTS

A total of 548 papers were analyzed on the basis of their titles and abstracts, and the following were excluded: 20 technical protocols; 6 reviews; 231 papers that addressed genetic diversity of pathogens, rather than genetic diversity of patients; 198 papers that did not study sepsis as a relevant outcome; 59 studies that comprised age groups other than only neonates; 7 studies that did not address genetic polymorphisms; 2 case reports; 1 study that did not evaluate humans; 1 paper written in Polish, with no English translation; and 1 study that was not available. The remaining 22 articles were fully analyzed^(9,)(21)-(41)^(Table 4). Their contents were summarized in the following sections, which address different SNPs. In several studies addressing more than one specific polymorphism, the information on each polymorphism is summarized with emphasis proportional to the degree of statistical and epidemiological relevance, as well as to the attention it received in previous studies.

**Table 4:** Scientific literature subjected to in-depth review

Author	Year	Country	Patients	Controls	Findings
Aydemir et al.^21^	2011	Turkey	31 preterm infants with fungal septicemia	30 preterm infants who had no invasive fungal infection	No association between codon 54 (B allele) polymorphism in exon 1 of the MBL gene and massive fungal sepsis
Hartel et al.^9^	2011	Germany	Cohort 1: 344 septic VLBW infants; Cohort 2: 555 septic VLBW infants	Cohort 1: 1600 non-septic VLBW infants; Cohort 2: 371 non-septic VLBW infants	No association between late onset sepsis and the SNP TNF- 308 G/A
Abu-Maziad et al.^22^	2010	USA	79 preterm infants with sepsis	202 preterm infants without sepsis	TLR2, TLR5, IL-10 and PLA2G2A polymorphisms predispose to sepsis in preterm infants
Auriti et al.^23^	2010	Italy	42 neonates with sepsis	85 neonates without infection	No association between the MBL2 genotypes and sepsis
Koroglu et al.^24^	2010	Turkey	Proven sepsis: 11 preterm newborns; Clinical sepsis: 42 preterm newborns	57 preterm infants with no sepsis	No association between MBL polymorphism and culture-proven sepsis; however, a risk of clinical sepsis was shown
Spiegler et al.^25^	2010	Germany	706 septic VLBW infants genotyped for ACE-ins/del genotype; 709 septic VLBW infants genotyped for ATR-1166A/C genotype	503 non-septic VLBW infants genotyped for ACE-ins/del genotype;495 non-septic VLBW infants genotyped for ATR-1166A/C genotype	No impact of any renin-angiotensin system SNPs; no association was found between the ATR-1166A/C genotype or ACE-ins/del genotype and neonatal sepsis
Abdel-Hady et al.^26^	2009	Egypt	54 septic full-term neonates	70 non-septic matched full-term neonates	The IL-6 -174 and IL-10-1082 genotypes were not significantly different in neonates with bloodstream infections, compared with controls
Bertalan et al.^27^	2008	Hungary	22 septic preterm neonates	103 non-septic preterm neonates	BclI, N363S and ER22/23EK polymorphisms of the glucocorticoid receptor gene were not associated with risk of sepsis
Reiman et al.^28^	2008	Finland	11 septic preterm VLBW infants	96 non-septic preterm VLBW infants	Association between the IL-6-174 CC genotype and increased sepsis prevalence in VLBW infants
Dzwonek et al.^29^	2008	England/ Poland	47 septic preterm infants	111 non-septic preterm infants	Analyses of the effect of MBL genotype in newborns of gestational age < 28 weeks and birth weight < 1000 g did not show a statistically significant association with sepsis
van der Zwet et al.^30^	2008	Holland	Low MBL genotype: 8 septic neonates; Medium MBL genotype: 15 septic neonates; High MBL genotype: 41 septic neonates	Low MBL genotype: 30 non-septic neonates; Medium MBL genotype: 60 non-septic neonates High MBL genotype: 44 non-septic neonates	No relationship was found between MBL genotype and the risk of nosocomial sepsis
Schueller et al.^31^	2006	Germany	67 preterm infants (< 32 weeks of gestation) and early-onset sepsis	102 healthy newborns born after 32 weeks of gestation	No association between polymorphisms of TNF-308 and LTA +252 and early-onset sepsis in newborns with < 32 weeks of gestation.
Derzbach et al.^32^	2006	Hungary	36 septic LBW infants	89 non-septic LBW infants	No association was found between the polymorphisms E-selectin Ser128Arg, P-selectin Thr715Pro, L-selectin Pro213Ser and the risk of sepsis
Hartel et al.^33^	2006	Germany	198 septic VLBW infants	1008 non-septic VLBW infants	Higher rate of sepsis for carriers of factor XIII Val34Leu SNP
Baier et al.34	2006	Canada	148 septic VLBW infants	145 non-septic VLBW infants	The IL-6 -174C allele was associated with increased incidence of late bloodstream infection (BSI) in AA but not in Caucasian infants. The IL-10 -1082A allele was associated with increased incidence of late BSI. The CD14 -260 C/T SNP did not alter the overall risk of BSI in ventilated VLBW infants.
Göpel et al.^35^	2006	Germany	97 VLBW infants who evolved with sepsis	320 non-septic VLBW infants	No association between sepsis and IL-6-174 genetic polymorphism
John Baier et al.^36^	2005	Canada	149 septic VLBW infants	146 non-septic VLBW infants	The ACE I/D polymorphism did not have a significant effect on incidence of sepsis
Hedberg et al.^37^	2004	USA	82 mechanically ventilated VLBW infants with late bacteremia	91 mechanically ventilated VLBW infants without late bacteremia	The TNF-308 G/A SNP had no influence on the incidence of either early or late bacteremia or fungemia
Ahrens et al.^38^	2004	Germany	50 septic VLBW infants	306 non-septic VLBW infants	VLBW infants carrying the NOD2-3020ins C allele (n = 15) and the IL-6-174 G allele (n = 121) had a significantly higher rate of blood culture-proven sepsis.
Bessler et al.^39^	2004	Israel	34 septic preterm LBW infants aged 24-35 weeks of gestation	61 non-septic preterm LBW infants aged 24-35 weeks of gestation	No impact of IL-1ra genetic polymorphism on early onset sepsis
Harding et al.^40^	2003	England	51 septic preterm infants aged < 32 weeks of gestation.	106 non-septic preterm infants aged < 32 weeks of gestation.	Increased confirmed bacteriological sepsis with IL-6-174 GG allele
Treszl et al.^41^	2003	Hungary	33 septic VLBW neonates	35 non-septic VLBW neonates	TNF-308, IL-1β, IL-4 receptor α chain, IL-6 and IL-10 genes are not risk factors for sepsis in LBW infants

VLBW = very low birth weight; LBL = low birth weight; BSI = bloodstream infection; MBL = mannose-binding lectin; TNF = tumor necrosis factor; IL-6 = interleukin 6; IL1β = interleukin 1β; IL-4 = interleukin 4; TLR2 = toll-like receptor 2; TLR5 = toll-like receptor 5; ACE I/D = angiotensin converting enzyme insertion/ deletion; ATR = angiotensin II type 1 receptor; PLA2G2A = phospholipase A2, group IIA; LTA = lymphotoxin alpha; SNP = single nucleotide polymorphism.

### TNF-alpha gene polymorphism

We retrieved four studies addressing the association between the SNP TNF-308 and development of neonatal sepsis. A study carried out on a small cohort of 103 neonates did not show any association between the SNP TNF-308 and neonatal sepsis. ^41^ Similar negative results were found in two additional studies. The first included 173 VLBW neonates who underwent mechanical ventilation, ^37^ while the other ^31^ compared the frequency of this genotype among 61 premature neonates (< 32 weeks) with early onset sepsis and among 102 healthy neonates (> 32 weeks). However, in the first study by Hedberg et al., the septic patients presenting AA/GA genotypes had a functional studies; b) stringent criteria for sepsis (microbiologimortality rate from sepsis that was three times greater than cally-proven); and c) confirmation of Hardy-Weinberg equilibamong those presenting the GG genotype. ^37^ rium. Its limitations were: a) no assessment of ethnicity; and b) limited microbiological information.

The remaining doubts about the lack of association between the SNP TNF-308 and development of neonatal sepsis, which had persisted mainly due to the limited sample size of the studies carried out earlier, were subsequently ruled out by a study on 2870 VLBW infants. ^9^ This study had the following strong points, besides the large cohort size: a) genotyping supported byfunctional studies; b) stringent criteria for sepsis (microbiologically-proven); and c) confirmation of Hardy-Weinberg equilibrium.Its limitations were: a) no assessment of ethnicity; and b)limited microbiological information.

Thus, although being a carrier of the SNP TNF-308A was shown to increase the risk of sepsis in a meta-analysis that included mostly adults and older children, ^44^ there is so far no evidence of any association between the SNP TNF-308 G/A and increased risk of sepsis during the neonatal period.

### Interleukin-6 gene polymorphism

Six studies addressing the association between the SNP IL-6-174 and neonatal sepsis were identified. Two of them showed that there was a small risk associated with the SNP IL-6-174 C allele and neonatal sepsis. The first study ^40^ included 157 neonates (< 32 weeks) predominantly composed of Caucasians (92%) and showed that there was a risk of neonatal sepsis associated with the SNP IL-6-174 GG (odds ratio, OR = 2.7; P = 0.01; in comparison with GA/AA). The strong points of this study included: a) very stringent criteria for septicemia (which may well have contributed to the strong effect detected); b) Hardy-Weinberg equilibrium and ethnicity evaluation; and c) correction for bias of multiple siblings. The limitations were essentially due to the small cohort size. Similar results were found by Ahrens et al. ^38^ in a study that included 356 VLBW Caucasian infants and showed that there was a risk of neonatal sepsis associated with the SNP IL-6-174 GG (OR = 0.19; P = 0.03). This study also showed that the higher rate of sepsis in this group reflected higher infection rates due to Gram-positive organisms, and that sepsis was not detected in patients who had been given prophylaxis using teicoplanin.

Apparently contradictory results were seen by Baier et al. ^34^ in a study that included 293 VLBW infants (< 1500 g; comprising 233 African-American, 57 Caucasian and three Latin-American infants) who underwent mechanical ventilation. This study showed that the IL-6-174 C allele was associated with increased incidence of sepsis. However, this effect was observed only in African-American subjects and not in Caucasian subjects. With a small cohort of 33 septic, 35 infected and 35 healthy VLBW infants, Treszl et al. ^41^ also failed to detect an association. Another study, with a large cohort that included 1206 VLBW preterm infants, mostly Caucasian, was equally unable to show any association between the SNP IL-6-174 and neonatal sepsis. ^35^ The strong points in this study included: a) large cohort size; b) careful microbiological characterization of bloodstream infections and colonization; and c) stringent criteria for sepsis. Recently, data from these five studies were consolidated into a meta-analysis ^45^ that showed that the SNP IL-6-174 had a modest carrier C effect in relation to neonatal sepsis (RR 0.9; 95% CI 0.62-1.31). 

After this meta-analysis, Reiman et al. ^28^ carried out a study that included 107 VLBW infants and showed that a risk of sepsis was associated with the SNP IL-6-174 CC (CC versus (vs) GG: OR = 3.05 P = 0.15; CC vs GC: OR = 18.27, P = 0.01). Although this Finnish study did not describe the ethnicity of the population included, the only other population for which an increased risk of sepsis was demonstrated in association with genotype CC consisted of African-Americans, ^34^ who are probably not representative of the population studied by Reiman et al. ^28^ It therefore remains to be seen whether ethnicity plays a role in the apparent discrepancies between the data from Reiman et al. and from other studies. In another study on an Egyptian population that included 54 fullterm neonates presenting bloodstream infections and 70 matched full-term neonates, Abdel-Hady et al. also found no association between the SNP IL-6-174 and neonatal sepsis. ^26^

Therefore, the real association presented by the SNP IL-6174 with neonatal sepsis development remains an open question, even for a particular ethnic group, apart from African-Americans. Further studies are necessary with a larger sample size. Two points should be considered in future studies. The first concerns the importance of description and, if possible, assessment of genotypic stratification according to ethnicity. The second concerns the importance of including the microbiological identification of the agent in the sepsis definition, since there is evidence that the SNP IL-6-174 may be related to an increased risk of sepsis caused by Gram-positive organisms.

### Mannose-binding lectin polymorphism

Mannose-binding lectin (MBL) is a human collectin and an important protein in the humoral innate immune system. MBL is a circulating pattern-recognition molecule that recognizes carbohydrate structures on the surface of a wide range of microorganisms, including bacteria, viruses, yeasts, protozoa and multicellular parasites, and thereby provides first-line defense. It also activates the complement system through a distinctive third pathway, independent of both antibody and C1 components.^(28),(46)^

Our search retrieved five studies that addressed the impact of MBL polymorphism and neonatal sepsis, but none of them identified any association between this genetic polymorphism and neonatal sepsis. A study that included 742 neonates did not find any association between MBL2 gene polymorphism and neonatal sepsis. ^30^ The strong points of this study were firstly its large cohort size and secondly good microbiological characterization of sepsis cases. Interestingly, the authors suggested that the possible cause of the lack of association could be a reflection of the predominance of bloodstream infections due to coagulase-negative staphylococci. MBL is not very helpful against these organisms.

Similar negative results were found in another study carried out on 158 preterm infants. ^29^ In this study, predominance of infection by coagulase-negative staphylococci was also observed. One important finding was the demonstration that MBL level, low birth weight and low gestational age were independently associated with the risk of sepsis. The probability of sepsis in neonates of < 28 weeks or < 1000 g with MBL levels < 400 ng/ml was found to be 70% in that study; in contrast, for those with MBL levels above 400 ng/ml, the risk of sepsis was 47%.

Auriti et al. ^23^ also showed, in a prospective study on 365 neonates, that the median MBL concentration on admission was significantly lower in infected than in non-infected neonates (P < 0.001). Moreover, they showed that lower levels of MBL on admission were associated with an increased risk of infection, independently of gestational age and invasive procedures. Another interesting finding in this study resulted from stratification of the neonates according to the pathogens isolated in blood cultures. Lower levels of MBL were significantly associated with a risk of sepsis due to Gram-negative organisms (OR = 0.58; P < 0.005), but not due to *Candida* species (OR = 0.56; P = 0.97) or Gram-positive organisms (OR = 0.56; P = 0.20). 

Despite highlighting the importance of MBL, a genotyping analysis carried out on 127 neonates failed to show any associationbetween MBL2 genetic polymorphism and neonatal sepsis. ^23^

Consistent with the lack of association between MBL levels and infection by *Candida* sp observed in the study by Auriti et al., another study on a small cohort of 30 septic neonates and 30 healthy premature neonates did not find any association between MBL2 gene polymorphism and nosocomial invasive fungal infection. ^21^ Negative results were also found by Koroglu et al. ^24^ on 99 premature neonates, in which sepsis was defined by microbiological identification of pathogens in blood cultures. However, when clinical sepsis was the outcome, the frequency of sepsis was higher in the group of infants with MBL gene polymorphism than among infants with wildtype MBL genotype (61.2 versus 31.7%, respectively, P = 0.008). 

Thus, there is so far no evidence that MBL2 gene polymorphism is associated with neonatal sepsis. ^24^ One important issue raised by Korogulu et al. is the definition of sepsis. Although sepsis definition based on microbiological identification provides greater certainty for diagnosing sepsis, the low sensitivity of blood cultures and high prevalence of antibiotic usage among neonatal intensive care unit patients can lead to difficulties in microbiological identification. Thus, since finding a positive blood culture is a requirement for sepsis definition, septic patients will certainly be found to be positive. On the other hand, many patients with negative blood cultures and who are considered to be nonseptic may present criteria for clinical sepsis, which may introduce bias into the analysis. Future studies should therefore conduct analyses for both clinical sepsis and proven sepsis.

### IL-1ra gene polymorphism

IL-1 receptor antagonist (IL-1ra) is a naturally occurring competitive inhibitor of the proinflammatory cytokine IL-1. Several studies have shown that carrier status for allele 2 of the IL-1ra (IL-1raA2) SNP is associated with greater production of IL-1ra and IL-1β *in vivo* and *in vitro*.^(47)-(49)^ The single study identified in our search ^39^ was carried out on 95 premature neonates (24-35 weeks of gestational age) and, despite confirming that IL-1ra gene polymorphism had an impact on preterm delivery, it failed to document any impact on early-onset sepsis. The strong points of this study included: a) ethnicity clearly defined; b) gestational age carefully determined; c) a small difference in gestational age (29-32 weeks) between preterm and healthy infants, but reflected in clear differences in birth weight; d) good clinical and microbiological definition of sepsis; and e) incorporation of extra data from adults in an unrelated German study.

### Selectin polymorphism

Only one study addressing selectin polymorphism was retrieved. It included 125 low birth weight infants and 156 healthy term neonates and did not show any association between selectin gene polymorphism and neonatal sepsis, although L-selectin was shown to have an impact in relation to preterm birth. ^32^

### Angiotensin-converting enzyme insertion/deletion polymorphism

The renin-angiotensin system plays a complex role in the pathophysiology of sepsis. The angiotensin-converting enzyme (ACE) leads to production of angiotensin II, which is a potent vasoconstrictor and stimulates aldosterone secretion, thereby leading to retention of water and salt. More recently, some evidence of the expression of ACE by macrophages and T lymphocytes, as well as the finding that ACE is also upregulated in the inflammatory response, ^50^ have drawn attention to the inflammatory role of ACE. 

Our search retrieved two studies addressing neonatal sepsis and angiotensin-converting enzyme insertion/deletion polymorphism. The first of these included 295 mechanically ventilated VLBW infants and did not show any association between angiotensin-converting enzyme (ACE) insertion/deletion (I/D) polymorphism and neonatal sepsis. ^36^ Spiegler et al. confirmed previous observations on the lack of impact of polymorphisms in the renin-angiotensin system, in a large cohort of 1209 VLBW infants. ^25^

### Polymorphisms associated with glucocorticoid sensitivity

A single study addressing the association between neonatal sepsis and genetic polymorphisms that affect sensitivity to glucocorticoids (BclI, N363S and ER22/23Ek gene polymorphism) was identified. This study was carried out among 125 premature neonates (28-35 weeks) and did not show any association between these three gene polymorphisms and neonatal sepsis. ^27^

### Polymorphisms affecting genes of hemostatic factors

A German multicenter study prospectively evaluated the impact of SNP in relation to genes that code for hemostatic factors (Leiden Factor V; prothrombin G20210A; Factor VII -323 del/ins; and Factor XIII Val34Leu) in 586 very low birth weight newborn children. This analysis was complemented by a retrospective study on a second, comparable group of 595 newborn children. ^33^ Carriers of the Val34Leu substitution in Factor XIII presented a higher rate of sepsis and longer hospitalization, in comparison with non-carriers. On the other hand, 323del/ins polymorphism of the Factor VII gene behaved as a potential protective factor against bronchopulmonary dysplasia. 

### Alternative approaches to the impact of SnP on neonatal sepsis

In a retrospective case-control study on 535 preterm infants, a total of 49 distinct SNPs affecting 19 separate candidate loci, including genes coding for inflammatory cytokines (IL-6, IL-10, IL-1β and TNF), cytokine receptors (IL-1RN), *toll-like* receptors (TLR2, TLR4 and TLR5) and endotoxin-binding proteins (CD14) were genotyped. ^22^ The subjects were stratified into three groups (sepsis, suspected sepsis and controls) and genotypes were correlated through a transmission disequilibrium test. The authors of this study report that birth weight, gestational age, time elapsed from membrane rupture and presence of clinical chorioamnionitis were strongly associated with sepsis. SNPs in the genes for TLR2 (rs3804099), TLR5 (rs5744105), IL-10 (rs1800896), and PLA2G2A (rs1891320) were associated with sepsis. Allelic variants in PLA2G2A and TLR2 were associated with Gram-positive infection, while IL-10 was associated with Gram-negative infection. The authors concluded that the allelic variants PLA2G2A, TLR2, TLR5 and IL-10 could influence the risk of sepsis among preterm infants. The main limitation of this study lies in its approach, which differs in important ways from that taken in most other studies reviewed above. Hence, while its results are interesting and the approach may hold some promise, the lack of other studies using the same methodology, and the difficulty of reconciling the data with those obtained through the classical approach should emphasize the need for caution in transposing the conclusions to the clinical setting before confirmatory evidence has been obtained.

## DISCUSSION

Any attempt to screen newborns at intensive care units for a given polymorphism because of associations with sepsis relies on a number of assumptions: a) that the polymorphisms are associated with sepsis and its outcomes in such a way that makes genotyping an important part of the decision-making process as to whether aggressive antimicrobial therapy should be instituted; b) that this association is not contingent on ethnicity, complex haplotype definitions or the nature of the pathogens involved; and c) that the cost and labor involved in obtaining this genetic information are justified by the benefits expected. 

Contrary to the original expectations of many investigators, ^40^ ^,^ ^51^ a decade of efforts by many groups ^18^ ^,^ ^52^ ^,^ ^53^ to define one or more gene polymorphisms that are sufficiently predictive of susceptibility to sepsis and outcomes to warrant routine use in the neonatal intensive care unit has met with partial success at best. ^9^ ^,^ ^45^ A large proportion of the studies describe nega-tive results (see Results), even in the case of genes for which an impact on sepsis has been consistently reported among adults and older children. ^9^ ^,)(^ ^36^ Although some studies have reported significant associations between neonatal sepsis and gene polymorphisms, these results have been inconsistent with those from other groups at different times and/or with different populations of neonates (see Results). Most often, strong associations seen in initial studies on small or medium-sized cohorts were not confirmed by studies with much larger groups of patients (comparing ^51^ ^,)(^ ^54^ with ^9^ for TNF-α; and ^40^ with ^35^ for IL-6). There have been no instances in which a large cohort study was the first to identify a major effect on susceptibility to sepsis from any of the candidate genes, although strong effects on predisposing conditions such as premature birth39 could be identified through this approach. 

Importantly, this stalemate is not dependent on the exact methodology used. For example, one study ^22^ used a family-based approach (transmission disequilibrium test) to identify multiple candidate genes associated with neonatal sepsis in a relatively large cohort of infants, and was able to detect moderate to borderline associations with a number of genes (PLA2, TLR subtypes and IL-10). Additional strategies used the definition of septic "phenotypes" based on multiple parameters to improve sensitivity, taking care to correct for the effect of multiple comparisons. None of the most frequently studied polymorphisms in other studies (TNF or IL-6) made it into the list of promising candidates, although PLA2, TLR and IL-10 all fall within the category of immune and inflammatory genes. It is unclear, however, whether a strategy that was developed to explore genotype/phenotype relationships is appropriate for sepsis, which cannot rigorously be considered to be a "phenotype", since it is a rapidly evolving clinical condition, entirely dependent on infectious exposure that can neither be controlled, nor ruled out. 

One important aspect of these studies involves the ethnic diversity of the populations examined. In at least one well-controlled study, ^34^ ethnicity played a major role in modifying associations between polymorphisms in immunologically relevant genes (IL-6 and CD14) and sepsis-related outcomes. Ethnicity is an issue in other studies conducted in Israel, ^39^ the USA37 and Europe.^(3,)(25)^ In the latter, this issue is reflected through recent immigration from African and the Middle East25 or comparison between widely separated Caucasian populations. ^3^ Interpretations on genotyping data are likely to be affected by the ethnic makeup of the population of interest, and this represents a limiting factor for applying this approach to infants worldwide.

Another major issue is the complexity of the polymorphisms involved, which may be quite variable, from single nucleotide polymorphisms in the case of TNF-alpha ^8^ to complex insertion and deletion events in the case of ACE. ^36^ The complexity is highest in the case of MBL, ^34^ since the known mutations often prevent the polymerization that is required for generating a functional protein. Furthermore, such mutations are in strong linkage disequilibrium, and therefore determination of their frequencies is not a simple matter, but involves screening for the existing haplotypes, which do not correspond to all the theoretical possibilities.

This issue further complicates the already complex problem of the functional impact of polymorphisms. The entire rationale behind determining genotypes and allele frequencies depends on a significant connection between genome and function, in addition to the undisputable fact that possession of a functioning gene is necessary for production of a functional protein. In a very few cases, this connection is wellestablished. For instance, demonstration that possession of a given allele in the TNF-alpha system results in significantly reduced transcription rates and decreased protein release was achieved in a non-physiological ex vivo assay. ^51^ While it is possible that this reflects physiological potential in the context of sepsis, the latter is much more difficult to ascertain. Other related problems concern the relative impact of single polymorphisms in the TNF region of the MHC on both TNF-α and TNF-β; ^31^ and the interrelated effects of polymorphisms in IL-1RA on production of IL-1RA and of IL-1β. ^39^

One especially entangled situation concerns MBL.^(24,)(29)^ This protein presents a series of polymorphisms that interfere with assembly of functional multimers. As a consequence, individuals who are homozygous for any given mutation, or heterozygous for two distinct mutations, present severe deficiency in this innate microbicidal factor. However, heterozygotes possessing one wildtype allele may produce functional MBL multimers and, since exposure to pathogens stimulates MBL production, deficiency may be difficult to demonstrate. Here, more than anywhere else, the dissociation between genotype and its phenotypic effects is a major issue. Indeed, the clinically relevant issue is not which alleles an infant possesses, but how much functional MBL is produced. The discrepancies in the results between studies carried out in ethnically diverse populations^(24,)(29,)(30)^ may be difficult to reconcile without systematic evaluation of the circulating MBL levels. However, there is a good side to this, because measurement of MBL may eventually prove to be a better predictor of sepsis than genotyping and, as a corollary, MBL replacement therapy should be helpful in individuals lacking it. One important issue that cannot be settled at present is whether MBL administration to infants who are utterly lacking in functional multimers might induce antibodies to the exogenous protein. 

## CONCLUSION

Genotyping of polymorphisms in the context of neonatal sepsis should be focused on a few markers, in order to reduce labor and costs, and to maximize speed, which is essential in the intensive care setting. While target polymorphisms should be strongly associated with the outcome, in most or all susceptible neonates, the extant literature shows that critical reevaluation of this approach is necessary, since many genes have an impact on sepsis, but none of them meets these requirements. Most importantly, genotyping more than a few of them would be impractical, since their relevance to the decision-making process is difficult to determine. We suggest, instead, that once candidate polymorphisms have been identified in epidemiological studies, their functional relevance should be established, before recommendations for their clinical routine use as genotypic markers are made.
